# Supplementary material for: Poly(l-lactide-co-caprolactone-co-glycolide)-Based Nanoparticles as Delivery Platform: Effect of the Surfactants on Characteristics and Delivery Efficiency
Source: Nanomaterials (Basel). 2022 May 3;12(9):1550. doi: 10.3390/nano12091550 (PMC9103935; doi:10.3390/nano12091550)
Supplement: Supplementary file 1 [file nanomaterials-12-01550-s001.zip › nanomaterials-1671975-supplementary.pdf]

nanomaterials

Supplementary Materials

Poly(L-Lactide-*co*-Caprolactone-*co*-Glycolide)-Based Nanoparticles as Delivery Platform: Effect of the Surfactants on Characteristics and Delivery Efficiency

Magda M. Rebanda [1,2], Simona Bettini [3], Laura Blasi [1,4], Antonio Gaballo [1], Andrea Ragusa [1,3], Alessandra Quarta [1,*] and Clara Piccirillo [1,*]

1 CNR Nanotec, Institute of Nanotechnology, Campus Ecotekne, Via Monteroni, 73100 Lecce, Italy; magda.rebanda@hotmail.com (M.M.R.); laura.blasi@cnr.it (L.B.); antonio.gaballo@nanotec.cnr.it (A.G.); andrea.ragusa@unisalento.it (A.R.)
2 Laboratório Associado, CBQF – Centro de Biotecnologia e Química Fina, Escola Superior de Biotecnologia, Universidade Católica Portuguesa, 4169-005 Porto, Portugal
3 Department of Biological and Environmental Sciences and Technologies, University of Salento, Via Monteroni, 73100 Lecce, Italy; simona.bettini@unisalento.it
4 Institute for Microelectronics and Microsystems, Campus Ecotekne, Via Monteroni, 73100 Lecce, Italy
* Correspondence: alessandra.quarta@nanotec.cnr.it (A.Q.); clara.piccirillo@nanotec.cnr.it (C.P.)

List of abbreviations:

AFM—Atomic force microscope

CTAB—Cetrimonium bromide

DCF—Dichlorofluorescein

DCFH—2′,7′-dichlorodihydrofluorescein

DCFH-DA—2′,7′-dichlorofluorescein diacetate

DDS—Drug Delivery System

DLS—Dynamic light scattering

DMEM—Dulbecco's Modified Eagle's Medium

DMSO—Dimethyl sulphoxide

DNA—Deoxyribonucleic acid

DOXO—Doxorubicin

EDTA—Ethylenediamine tetraacetic acid

EE—Encapsulation Efficiency

FBS—Serum fetal bovine

FDA—Food and Drug Administration

FTIR—Fourier Transform Infrared

LC—Loading Capacity

MCF7—Michigan Cancer Foundation-7

MTT—Tetrazolium salt Thiazolyl blue

NC-AFM—Non-contact mode AFM

NP(s)—Nanoparticle(s)

PBS—Phosphate-buffered saline

PCL—Poly-ε-caprolactone

PDI—Polydispersity index

PEG—Polyethylene glycol
PLA—Poly(L-lactic) acid
PLCG—Poly(L-lactide-co-caprolactone-co-glycolide)
PLGA—Poly(lactic-co-glycolic) acid
PVA—Poly(vinyl alcohol)
RE—Release Efficiency
RIPA—radioimmunoprecipitation assay
TGA—Thermogravimetric analysis

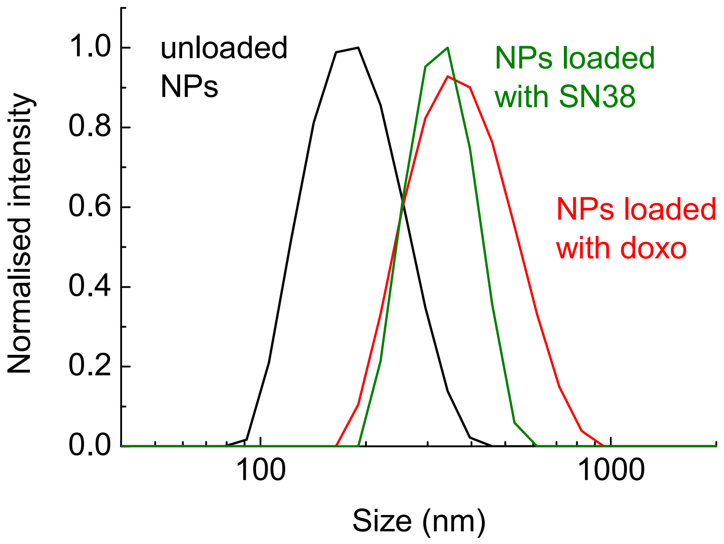

Figure S1. DLS data for NPs prepared using PVA/dextran as surfactants, both empty and loaded with DOXO.

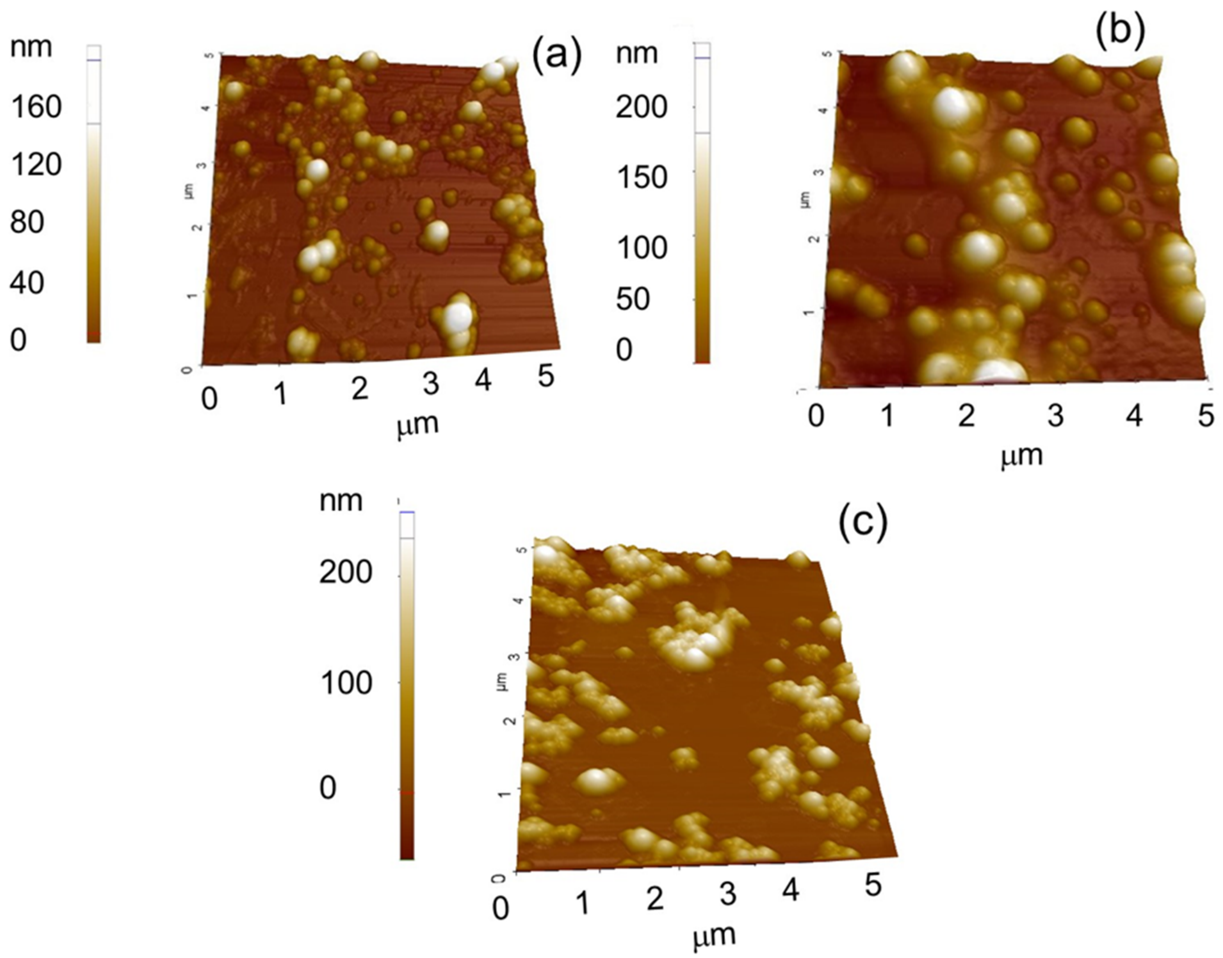

Figure S2. AFM micrographs for three types of NPs. (**a**) PVA, (**b**) DEX, (**c**) PVA/DEX.

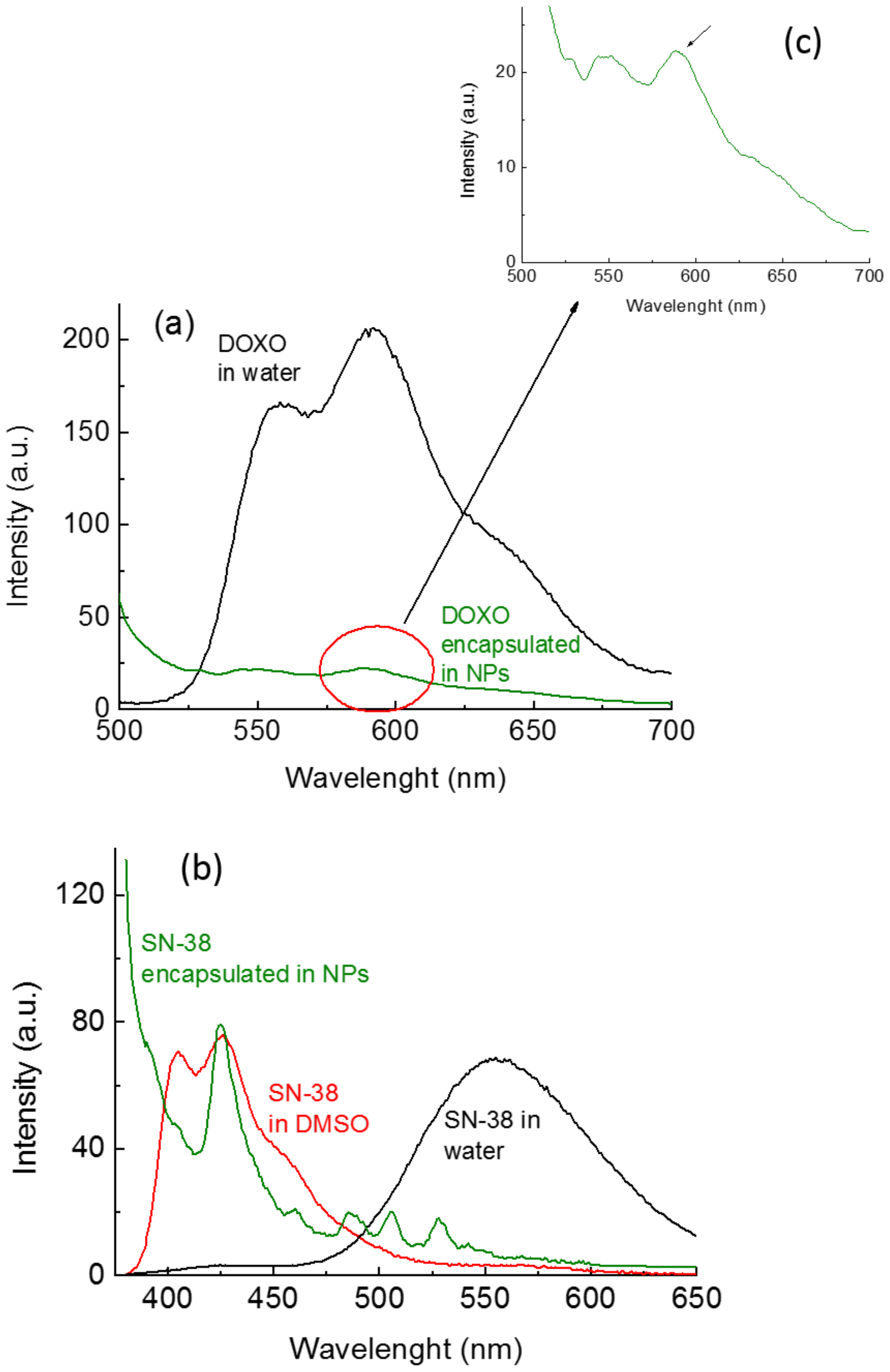

Figure S3. (**a**) Fluorescence spectra of free DOXO (black curve) and DOXO encapsulated into the NPs prepared with PVA/dextran (green curve), excitation wavelength 488 nm. (**b**) Fluorescence spectra of free SN-38 dissolved in DMSO and water (black and red curves) and of the drug encapsulated into the NPs prepared with PVA/DEX (green curve), excitation wavelength 370 nm. (**c**) The inset reports the curve of the encapsulated DOXO shown in (a) scaled up to show the two typical peaks of DOXO. The systems prepared with other surfactants showed similar features (data not shown).

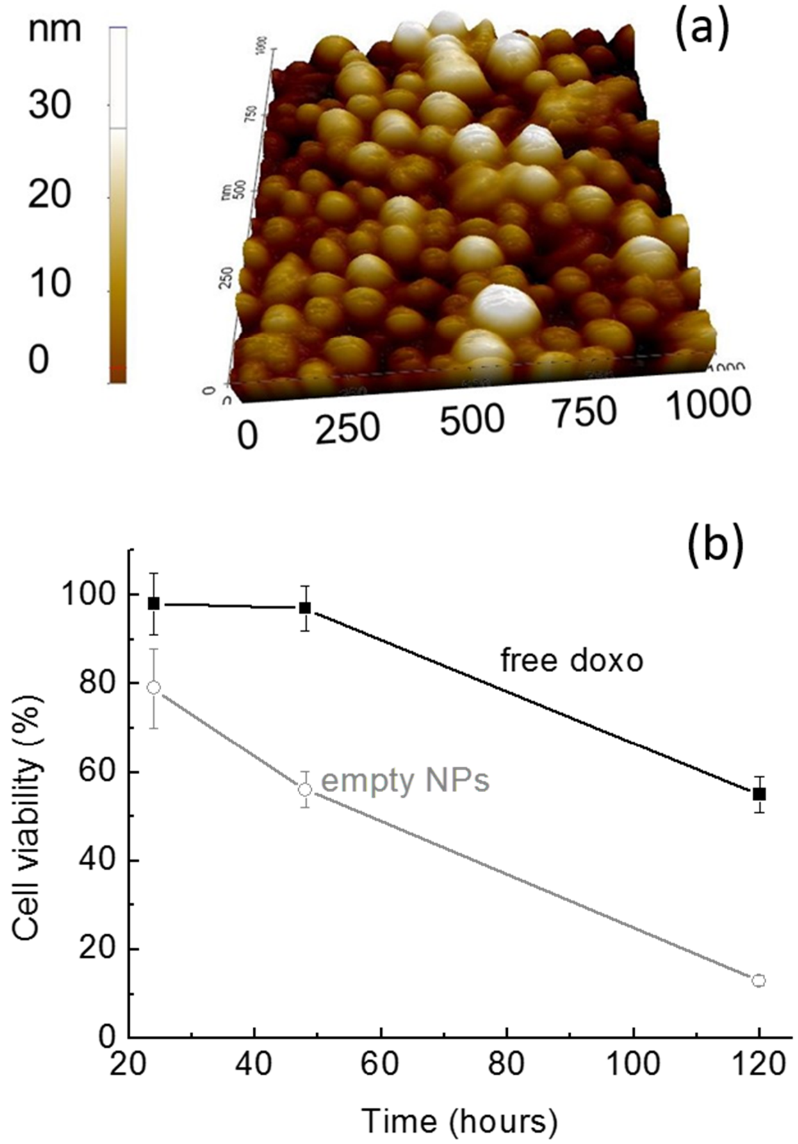

Figure S4. (**a**) AFM image and (**b**) cell viability for NPs prepared using CTAB as surfactant agent.

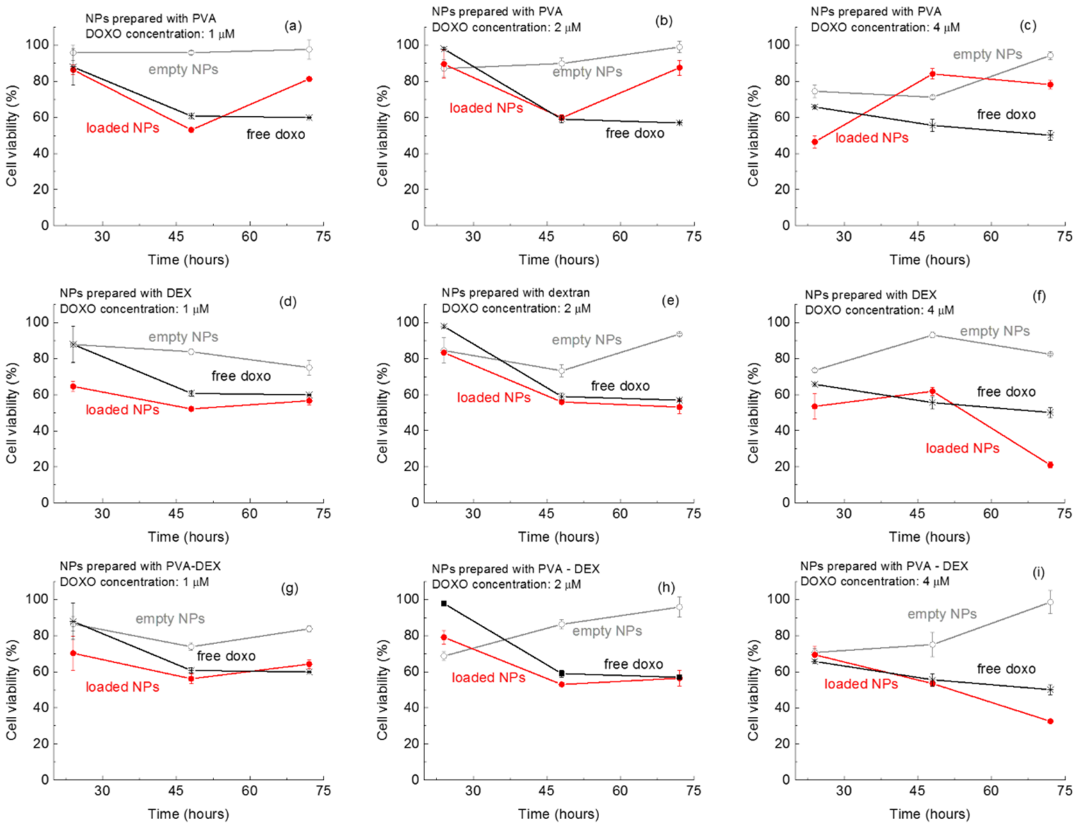

Figure S5. MTT viability tests of MCF7 cells incubated with the NPs coated with (**a–c**) PVA; (**d–f**) DEX and (**g–i**) PVA/DEX and loaded with DOXO. Three drug concentrations (1, 2 and 4 μM) and three time points (24, 48 and 72 h) were considered. The drug concentration points correspond to NP amounts ranging from 50 to 112 μg.

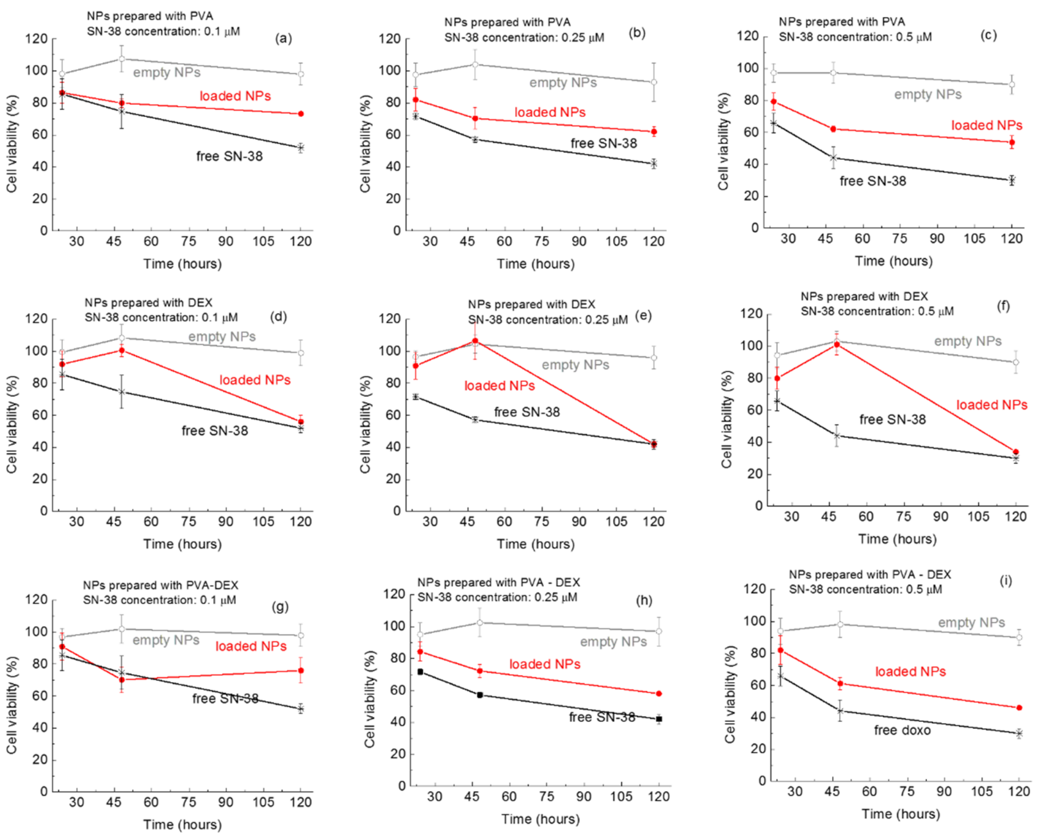

Figure S6. MTT viability tests of MCF7 cells incubated with the NPs coated with (**a**–**c**) PVA; (**d**–**f**) DEX and (**g**–**i**) PVA/DEX and loaded with SN-38. Three drug concentrations (0.1, 0.25 and 0.5 μM) and three time points (24, 48 and 120 h) were considered. The drug concentration points correspond to NP amounts ranging from 24 to 200 μg.

Table S1. Estimated NPs concentration (μg/mL) corresponding to DOXO concentrations of 1, 2, and 4 μM and SN-38 concentrations of 100, 200, and 500 nM.

	**DOXO**		
**Surfactant**	**1 μM**	**2 μM**	**4 μM**
PVA	50	100	200
DEX	25	50	100
PVA/DEX	28	56	112
	**SN-38**		
**Surfactant**	**100 nM**	**200 nM**	**500 nM**
PVA	24	48	120
DEX	20	40	100
PVA/DEX	40	80	200

Table S2. IC_{50} values of either empty or loaded NPs referred to the mass of nanoparticle (μg) needed to kill 50% of treated cells. The data have been determined after 72 h (in the case of DOXO) and 120 h (in the case of SN-38) incubation with MCF-7 cells. In each pair of samples (empty versus loaded NPs) the difference between the IC_{50} values is statistically significant ($p < 0.01$).

72 h incubation	IC 50 (μg NP)	120 h incubation	IC 50 (μg NP)
PVA	612 ± 53	PVA	454 ± 49
PVA with DOXO	164 ± 8	PVA with SN-38	107 ± 21
PVA/DEX	480 ± 24	PVA/DEX	450 ± 35
PVA/DEX with DOXO	51 ± 5	PVA/DEX with SN-38	89 ± 7
DEX	397 ± 36	DEX	349 ± 47
DEX with DOXO	79 ± 6	DEX with SN-38	82 ± 8
